# Controlling the Emotional Bias: Performance, Late Positive Potentials, and the Effect of Anodal Transcranial Direct Current Stimulation (tDCS)

**DOI:** 10.3389/fncel.2016.00159

**Published:** 2016-06-17

**Authors:** Florian Faehling, Christian Plewnia

**Affiliations:** ^1^Department of Psychiatry and Psychotherapy, Neurophysiology and Interventional Neuropsychiatry, University of TübingenTübingen, Germany; ^2^Werner Reichardt Centre of Integrative Neuroscience, University of TübingenTübingen, Germany

**Keywords:** cognitive control, late positive potential, transcranial direct current stimulation (tDCS), brain stimulation, negativity bias, working memory, EEG/ERP, neuromodulation

## Abstract

Cognitive control of emotional processing is essential for adaptive human behavior. Biased attention toward emotionally salient information is critically linked with affective disorders and is discussed as a promising treatment target. Anodal (activity enhancing) transcranial direct current stimulation (tDCS) has been shown to increase healthy and impaired cognitive control over emotional distraction and is therefore widely used for the investigation and experimental treatment of this disorder. In this study, event-related potential (ERP) were recorded parallel to tDCS to track its online effects. Healthy volunteers (*n* = 87) performed a delayed working memory paradigm with emotional salient and neutral distractors during stimulation with different intensities (sham, 0.5, 1, 1.5 mA). Measuring the late positive potential (LPP), an ERP that indexes attention allocation, we found that a valence-specific increase of the early portion of the LPP (eLPP, 250–500 ms) was associated with less emotional distraction in the sham group. Of note, stimulation with tDCS exerted an intensity related effect on this correlation. The later part of the LPP (lLPP, 500–1000 ms) was found to be correlated with reaction time, regardless of valence. General effect of tDCS on LPPs and task performance were not observed. These findings demonstrate that ERP recordings parallel to tDCS are feasible to investigate the neuronal underpinnings of stimulation effects on executive functions. Furthermore, they support the notion that the LPP induced by a distractive stimulus during a working memory task mirrors the additional allocation of neuronal resources with a specific sensitivity of the early LPP for highly arousing negative stimuli. Finally, together with the variable magnitude and direction of the emotional bias, the lack of systematic modulations of LPPs and behavior by tDCS further underlines the important influence of the individual brain activity patterns on stimulation effects both on the behavioral and neurophysiological level.

## Introduction

Cognitive control of emotional distraction is an important prerequisite for successful goal-oriented human behavior (Ochsner and Gross, 2005; Niendam et al., 2012). Its dysregulation plays a major role in the pathogenesis and maintenance of psychiatric disorders (Goschke, 2014). For instance, consistent with the neurocognitive model of depression (Warren et al., 2015) enhanced attention toward negative stimuli (“emotional bias”) represents a critical factor in the formation and perpetuation of depressive symptomatology and is therefore a promising, knowledge-based target of therapeutic interventions (Roiser et al., 2012). On a neural level, there is evidence that negatively biased processing derives from hypoactivation of prefrontal cortical regions, especially the dorsolateral prefrontal cortex (dlPFC), and a corresponding hyperactivity in deeper (e.g., limbic) brain areas (Dolcos and McCarthy, 2006; Groenewold et al., 2013; Korgaonkar et al., 2013; Clarke et al., 2014; Plewnia et al., 2015). Particularly, emotional distraction during the delay period in a working memory task has been linked with low dlPFC activity (Dolcos and McCarthy, 2006; Anticevic et al., 2010; Wessa et al., 2013). Consistently, in depressed subjects negative stimuli are associated with reduced activity in left dlPFC (Grimm et al., 2008; Groenewold et al., 2013).

Transcranial brain stimulation has been put forward as a promising option for the targeted modulation of biased cognitive processing (Plewnia et al., 2015). In particular, transcranial direct current stimulation (tDCS) is an easy to use, safe (Bikson et al., 2009) and well tolerated brain stimulation technique to modulate cortical activity by increasing (anodal tDCS) or decreasing (cathodal tDCS) the neuronal excitability (Nitsche et al., 2008).

Recently, we have demonstrated that anodal tDCS over the left dlPFC ameliorates the emotional bias in depressed subjects (Wolkenstein and Plewnia, 2013), whereas cathodal tDCS of this area can transiently induce a depression-like negativity bias in healthy volunteers (Wolkenstein et al., 2014). These findings may indicate a critical neurocognitive mechanism accounting for the treatment effects of brain stimulation on depression. A combination of stimulation and cognitive training may be a new method for targeted depression treatment (De Raedt et al., 2015; Plewnia et al., 2015). However, the neurophysiological mechanisms mediating effective control of emotional distraction as well as its association with tDCS effects and the dose-response relationship of stimulation still require further investigation. This is particularly important since inter-individual variability (López-Alonso et al., 2014; Wiethoff et al., 2014; Chew et al., 2015) and a non-linear effect of stimulation intensity (Batsikadze et al., 2013) often lead to inconsistent effects of electrical stimulation on neurophysiological, behavioral, and clinical parameters (Mondino et al., 2015).

The visually evoked late positive potential (LPP), an ERP recorded at midline electrodes, has been shown to be a marker of attention allocation toward salient emotional stimuli (Hajcak et al., 2013). It covers a relatively broad time-frame, beginning as early as 160 ms after stimulus onset, and remains present for several seconds (Hajcak et al., 2009). It is remarkably stable in contrast to autonomic markers such as skin conductance (Codispoti et al., 2006), is sensitive to emotional stimuli content (Flaisch et al., 2008; Weinberg and Hajcak, 2010a), task relevance (Olofsson et al., 2008; Weinberg and Hajcak, 2010b) and reliably exhibits higher amplitudes for emotional versus neutral stimuli in passive picture viewing paradigms (Schupp et al., 2000). Moreover, increasing cognitive demand, such as higher loads in a WM task, decrease the LPP induced by task-irrelevant, distracting stimuli, notably without specific influence of valence (MacNamara et al., 2011; Van Dillen and Derks, 2012; Schönfelder et al., 2014). Additionally, the LPP can be modulated by regulatory “top-down” mechanisms (Moratti et al., 2011) and has been discussed as a marker for emotion regulation (DeCicco et al., 2014). For instance, the instruction to suppress emotional reactions to negative pictures (Moser et al., 2006) reduces its amplitude. Critically, several studies have linked the LPP amplitude with task performance (Weinberg and Hajcak, 2011; Bamford et al., 2015). The neuronal basis underlying the complexity of the LPP seems to be a network of neuron populations in the occipital and parietal regions responsible for attention allocation as well as prefrontal cortex involved in higher cognitive manipulation of stimuli (Ochsner and Gross, 2005; McRae et al., 2010; Moratti et al., 2011; Sabatinelli et al., 2013).

In sum, the LPP seems a well-suited tool to examine the neurophysiology of cognitive control and the subtle modulatory effects of tDCS. One study assessed the effect of tDCS on a subsequent passive viewing paradigm of pictures, providing evidence for LPP modulation by electrical stimulation of the dlPFC (Hajcak et al., 2010a). Only a few studies have used a parallel tDCS-ERP design (Cunillera et al., 2015), as the current flow of tDCS complicates concomitant recording of electronic brain activity. However, this approach is critical, as it takes into account the influence of ongoing brain activity on tDCS effects (Antal et al., 2008; Zwissler et al., 2014; Benwell et al., 2015).

In the study presented here, we aimed at a further exploration of the neuronal mechanisms of cognitive control on negative distraction exemplified by LPPs, and the influence of anodal tDCS with different intensities using a parallel tDCS/ERP design in a delayed working memory task (DWM).

## Materials and methods

### Participants

We recruited healthy student volunteers via university announcements and email advertisement. Only female participants were included to improve homogeneity in the sample (Dolcos and McCarthy, 2006; Gardener et al., 2013). Participants received financial compensation for the experiment regardless of their performance. They were randomly assigned to one of the four stimulation conditions (sham, 0.5, 1.0, 1.5 mA tDCS). Written informed consent was obtained from all participants at the beginning of the study. The study was approved by the Ethics Committee of the Medical Faculty of the University of Tuebingen in accordance with the Declaration of Helsinki. Two participants had to be excluded due to protocol violations, five because of problems EEG registration during tDCS, and due to excessive noise in the EEG data (see electrophysiological data processing). In total, 87 participants were included in the analysis (sham: *n* = 22, 0.5 mA: *n* = 22, 1 mA: *n* = 22, 1.5 mA: *n* = 21). The German version of the Symptom-Checklist-90-R questionnaire (SCL-90-R, Franke, 2002) was used to screen for psychopathology. Furthermore, participants were asked to indicate whether they were smokers, drank coffee in the 2 h preceding the experiment, whether they used hormonal contraception and at what stage of their menstrual cycle they were at the day of the experiment. The Positive and Negative Affect Schedule (PANAS; Crawford and Henry, 2004) was completed by participants before and after the experiment to measure mood changes by DWM performance. It consists of 20 affective adjectives (10 positive and 10 negative) that are rated on a 4-point Likert scale. The Multiple Choice Vocabulary Test (MWT-B; Lehrl, 2005) was used to measure approximate general intellectual level. The Edinburgh Handedness Inventory (Oldfield, 1971) allowed for exclusion of left handed participants. A value below 50 was considered as an indicator for left-handedness. Finally, a computerized version of a digit span experiment as proposed by Sternberg (1966) was used as an approximate for a participant's working memory capacity.

### Study population characteristics

All participants were right handed (Edinburgh Handedness Inventory: *M* = 84.04, *SEM* = 1.91) healthy female (under)graduate students with normal or corrected to normal vision. Analysis of variance revealed that participants did not differ significantly across experimental groups in age [*M* = 23.85, *SEM* = 0.32, *F*_(3, 83)_ = 1.274, *p* = 0.289], percentage of smokers [*M* = 16.88%, *SEM* = 4.29, *F*_(3, 73)_ =0.227, *p* = 0.877], likelihood of coffee intake [*M* = 7.79%, *SEM* = 3.07, *F*_(3, 73)_ = 1.209, *p* = 0.313], day of the menstruation cycle [*M* = 12.64, *SEM* = 1.07, *F*_(3, 63)_ = 1.769, *p* = 0.162], usage of an oral contraception [*M* = 66.23, *SEM* = 5.43, *F*_(3, 73)_ = 0.545, *p* = 0.653] and MWT-B performance [*M* = 103.05, *SEM* = 1.24, *F*_(3, 83)_ = 0.57, *p* = 0.636]. Lastly, the four experimental groups did not differ in working memory performance as measured by digit span [*M* = 5.36, *SEM* = 0.14, *F*_(3, 83)_ = 0.603, *p* = 0.615].

### Experimental design

**Figure 1:**
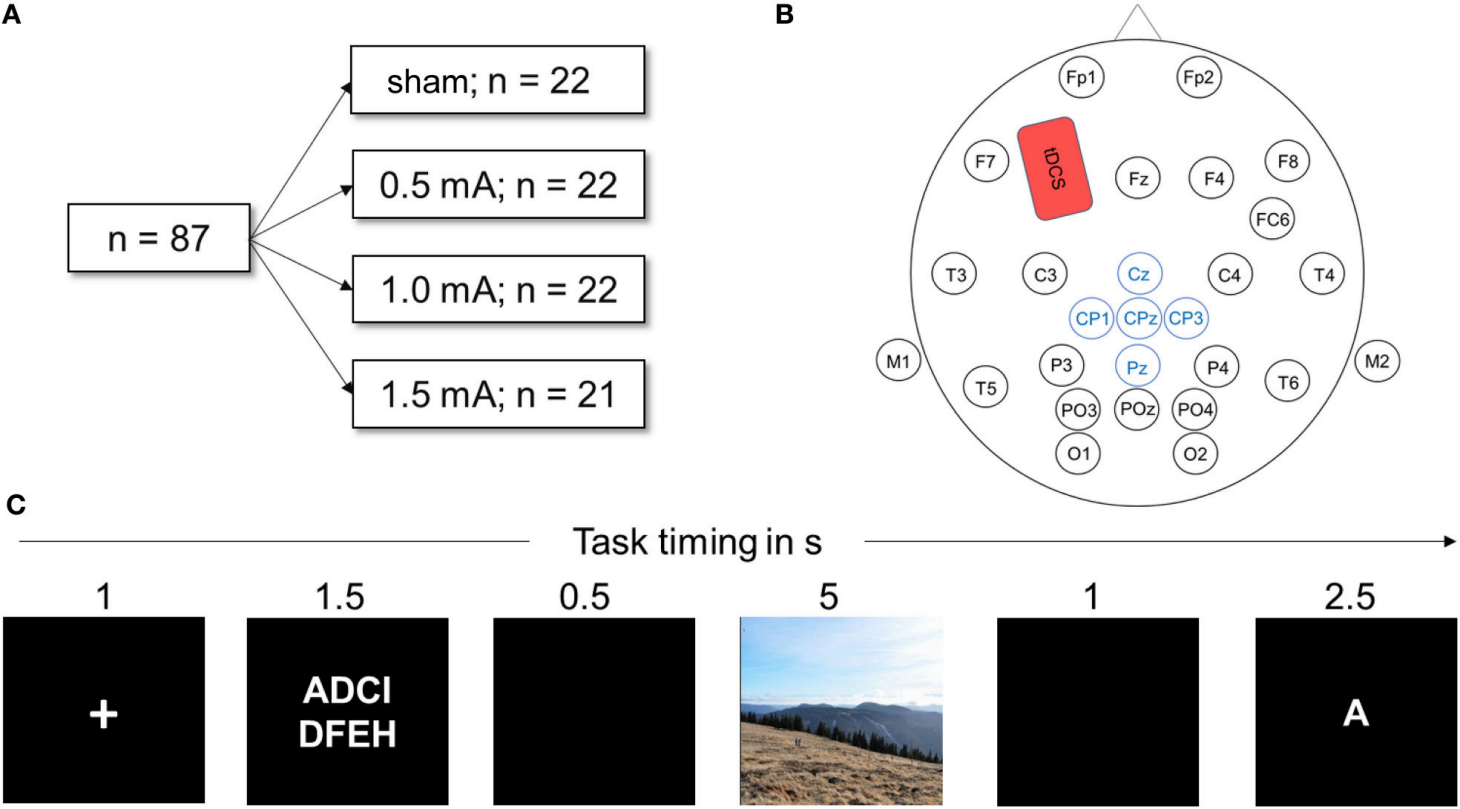
**(A)** Visualization of the experimental design. Eighty-seven healthy participants were randomly assigned to one of the four stimulation conditions. All performed the DWM. **(B)** Head map illustrating EEG and tDCS electrode placement used in this study. The anodal tDCS electrode (shown in red) was placed over F3, the cathode above the right deltoid muscle. Electrodes depicted in blue were averaged to obtain the LPP. **(C)** Schematic of one trial of the delayed working memory task (DWM). Eight letters (white on black ground) were presented and the participant had to memorize them. In a delay period, either a neutral or negative picture was shown. Next, a target letter was presented and the participant decided whether the letter was part of the string of eight letters by pressing a button. In total, the DWM comprised 80 of these trials.

In this single blinded, sham-controlled between-subjects design study, tDCS current intensity served as group variable with the levels sham stimulation, 0.5, 1, and 1.5 mA. Each participant performed one DWM session with parallel tDCS and EEG recording (see Figure 1A). Participants were naïve to the exact purpose of the study. They were handed an information sheet at the beginning of the task, which informed them that they would participate in an experiment about attention and emotional processing, that very negative pictures would be involved and that they either would be stimulated with an electrical current or would receive sham stimulation. Importantly, participants were not aware as to which experimental condition they belonged until after the experiment. Before they were informed whether they received stimulation, they completed a questionnaire to check if blinding was successful.

### Delayed working memory task

The delayed working memory task was displayed on a 21-inch TFT monitor; participants sat approximately 50 cm away from the screen. The EEG recording device was connected to the monitor via photo diodes to measure the exact time of stimulus onset. The task was implemented using Presentation software (Neurobehavioral Systems, Inc., Albany, CA) and consisted of 10 training trials followed by 120 experimental trials. Figure 1C depicts a sample trial from the DWM. At the beginning of the session, task instructions were given on the screen (see Appendix A for the exact text of the instructions). Each trial was preceded by a black screen inter-stimulus interval jittered randomly between 1 and 1.5 s was shown before every trial. Each trial then began with a white fixation cross displayed on a black background for one second. Next, a string of 8 letters was visible for 2.5 s, with white font on a black background. The letters were aligned in two rows of four letters and were selected randomly. A black screen was then displayed for 0.5 s. Next, a random picture of either negative or neutral content was shown for 5 s. Pictures filled the whole screen, displayed in color and each was shown only once for each participant. Next, a black screen appeared for 1 s. Then, a target letter was presented and the participant had to indicate whether the letter was part of the previously presented string by pressing the “f” key for “no” and the “j” key for yes on a commercial QUERTZ keyboard. Participants were always asked to use their right index finger to press “j” and the left to press “f,” to avoid confounding effects of laterality. One trial lasted for 12–12.5 s. A trial was assessed as correct if the participant correctly identified whether or not the letter was part of the string seen before within the time window of 2.5 s. After every 10th trial, a black screen was shown for 15 s to let participants relax.

### Stimulus material

Pictures were taken from the International Affective Picture Scale (IAPS) database (Lang et al., 2008) and normative ratings for the pictures were obtained from the online database of the IAPS. For the negative category, 40 highly arousing (*M* = 6.708, *SD* = 0.728 on a 1–9 scale where the higher the number, the higher the arousal rating) pictures with highly negative valence ratings (*M* = 1.59, *SD* = 0.325, on a 9-point Likert scale where the lower the number, the more negative the rating) were selected. Respectively, 40 pictures with low arousing ratings (*M* = 3.528, *SD* = 0.493, rated on a 9-point Likert scale ranging from 1 “very negative” to 9 “very positive”) and medium valence ratings (*M* = 5.067, *SD* = 0.722) were taken for the neutral category. For valence, negative pictures had significantly stronger negative ratings then neutral pictures [*t*_(78)_ = −26.302, *p* < 0.001]. Ratings for arousal also differed significantly with negative pictures rated more arousing than neutral pictures [*t*_(78)_ = 21.4882, *p* < 0.001]. Only scenes containing humans were used and the number of humans was matched between the categories. The negative pictures consisted of scenes of mutilated bodies (or parts of bodies such as injured hands), neutral pictures consisted of portraits of humans or everyday scenes (e.g., the inside of a supermarket). For the 10 training trials, additional 10 neutral pictures containing scenes without humans were selected (see Appendix B for a list of the selected pictures). Pictures were manually equated for luminance in Adobe Photoshop for Microsoft Windows. After completion of the experiment, participants rated each picture for valence and arousal on a 9-point Likert scale. The letters used in the DWM were presented at random or each trial using all 26 letters of the Latin alphabet.

### Transcranial direct current stimulation (tDCS)

Except in the sham-group, anodal tDCS was applied for the whole duration of the experiment with a battery driven stimulator (NeuroConn GmbH, Illmenau, Germany) via a pair plastic electrodes (35 cm^2^ surface area) that were connected to the skin with conductive paste. To target the left dorsolateral prefrontal cortex (dlPFC), the anodal electrode was placed on the scalp at F3 according to the international 10–20 system of electrode placement (see Figure 1B) and the reference electrode placed on the contralateral right deltoid muscle. The scalp electrode was placed under the elastic electrode cap and sufficiently fixated by the conductive paste. Stimulation was turned on after the training block and the task was started 1 min later. The current was ramped up and down for 10 s at the beginning and the end of stimulation. The impedance of the electrodes was always below 10 kΩ. The maximum duration of stimulation was 28 min, depending on the length of the randomly jittering pauses in between trials. For the sham condition, a current of 1 mA was ramped up and down for 10 s but only maintained for 30 s. This produces the same tingling sensation but does not induce sustaining effects on cortical activation (Ambrus et al., 2012; Palm et al., 2013).

### Electroencephalography (EEG) recording

Continuous EEG recording was performed according to standard procedure (Light et al., 2010) using an elastic cap (EASYCAP GmbH, Hersching, Germany) and the 32-channel EEG recordings system NEUROPRAX (NeuroConn GmbH, Illmenau, Germany). Twenty-five electrodes were fixed on the scalp according to international 10/20 system (see Figure 1B). The locations of the electrodes were carefully cleaned with alcohol and cotton swabs to minimize resistance. Mastoid electrodes were positioned beneath each earlobe on the mastoid bone. Eye-electrodes were positioned approximately 1 cm below and above the right eye for the vertical eye movement recordings and 1 cm to the right side of the right eye and the left side of the left eye for horizontal eye movement recordings.

### Behavioral data processing

Reaction time (RT) was measured as the time in milliseconds (ms) between picture onset and keypress. Reaction times that exceeded standard deviation (SD) by a factor of 2 were excluded. This lead to an exclusion of *M* = 6.21% (*SEM* = 0.29%) of trials containing negative and 6.47% (*SEM* = 0.37%) containing neutral pictures being rejected. The number of trials rejected did not vary significantly for valence [*F*_(1, 83)_ = 0.388, *p* = 0.563] or stimulation [*F*_(3, 83)_ = 0.382, *p* = 0.766] nor was there an interaction effect [*F*_(3, 83)_ = 1.312, *p* = 0.276]. Mean accuracy (AC) was calculated as the proportion of correct responses relative to the total number of responses.

### Electrophysiological data processing

EEG data analysis was performed with the MATLAB (MATLAB and Statistics Toolbox Release 2012a, The MathWorks, Inc., Natick, Massachusetts, United States) based EEGLAB toolbox (Delorme and Makeig, 2004) and the EEGLAB toolbox ERPLAB (Lopez-Calderon and Luck, 2014). Raw EEG data were referenced to an average of mastoid electrodes on the left and right side. Band-pass filters with a low and high cutoff of 0.1 and 35 Hz, respectively, and a notch-filter at 50 Hz were applied. Eyeblink correction was performed using the ICA-approach implemented in EEGLAB. Stimulus-locked trials were extracted ranging from −200 to 1000 ms relative to stimulus (i.e picture) onset. The 200 ms pre-stimulus time served as a baseline (Urbach and Kutas, 2006). A semi-automated artifact correction procedure was then used: Trials with a voltage step of >50 μV between sample points, >200 μV within a trial or >50 μV within a 100 ms window were excluded using ERPLAB algorithms. Data were visually inspected to remove remaining artifacts. One subject was excluded after artifact detection as >95% of trials were rejected by the algorithm. In the remaining sample, an average of *M* = 7.44% of trials for negative (*SEM* = 0.78%) and *M* = 7.59% for neutral (*SEM* = 0.85%) picture trials were discarded. In sum, 3221 negative and 3216 positive picture trials were included in the ERP analysis. The number of rejected trials did not vary significantly for negative vs. neutral pictures [*F*_(1, 83)_ = 0.051, *p* = 0.823]. A significant effect was found for stimulation [*F*_(3, 83)_ = 106.002, *p* = 0.007]. Table 1 shows the percentage of rejected trials across stimulation conditions.

**Table 1:** **Rejected EEG trials in percent (%) separately for valence and stimulation intensity**.

	**Total**		**Sham**		**0.5 mA**		**1 mA**		**1.5 mA**	
Negative pictures	7.44	(0.78)	5.57	(1.13)	6.70	(1.72)	5.45	(1.02)	12.26	(1.88)
Neutral pictures	7.59	(0.85)	4.66	(1.02)	7.84	(1.74.)	6.48	(1.39)	11.55	(2.26)

*Standard error of the mean (SEM) is shown in brackets*.

The LPP was scored as an average from five centro-parietal sites where it was maximal in earlier studies (Hajcak et al., 2009; MacNamara et al., 2011): Pz, CPz, Cz, CP1, and CP2. Following the literature on the time course of the LPP, we divided the LPP in two time windows, adjusting the limits following visual curve inspection: An early window (eLPP) from 250 to 500 ms and a late window (lLPP) from 500 to 1000 ms after picture onset were selected. Trials were separated for the two picture valence categories (negative, neutral). In that way, two ERP curves per participant were generated, each consisting of an average of up to 40 stimulus-locked curves. For analysis of picture valence differences, ERP curves of all participants were then averaged. To examine stimulation effects, ERP curves for each stimulation condition were generated. We measured the LPP using the mean amplitude measurement of ERPLAP, calculating the mean amplitude for eLPP between 250–500 ms and lLPP between 500–1000 ms (for a review of alternative measurements, see Luck, 2014).

### Data analysis

Statistical analyses was performed with SPSS Statistics for Microsoft Windows (version 22.0). To separately account for the influence of distraction and tDCS, data from the four experimental groups were analyzed in two steps. First, we examined the effects of picture valence on behavioral and electrophysiological measures in the sham group alone. Then, the influence of tDCS of different intensities was investigated by analysis of the complete sample comprising the sham, 0.5, 1.0, and 1.5 mA tDCS conditions.

Paired *t*-tests were performed on RT, AC, eLPP, and lLPP to examine differences between negative and neutral distractor trials for the sham-stimulated subjects. For the complete sample, a repeated measures analysis of variance (ANOVA) was conducted on RT, AC, eLPP and lLPP with valence (negative/neutral) as within-subjects factor and stimulation (sham, 0.5, 1, 1.5 mA) as between-subjects factor. To investigate the general association between DWM performance and brain activity, we correlated the LPP amplitudes and RT for negative and neutral distractors in the sham group and the complete sample.

Lastly, to examine valence specific effects, a difference score (Δ) was calculated for RT and LPP subtracting measures of trials with neutral pictures from trials with negative pictures (ΔRT_neg-neu_ = RT_neg-_−RT_neu,_ΔLPP_neg-neu_ = LPP_neg-_−LPP_neu,_). The relationship between the amount of distraction induced by the negative valence of pictures and the corresponding valence-specific brain activity was determined by the correlation between ΔRT_neg-neu_ and the ΔLPP_neg-neu_ (ΔeLPP_neg-neu_ and ΔlLPP_neg-neu_). These analyses were performed for the sham group and the complete sample including the different tDCS intensities.

## Results

### Mood changes

Participants' mood ratings, as reflected by the PANAS, changed significantly before and after DWM task performance. After the experiment, mood was rated less positive [before: *M* = 31.02, *SEM* = 0.63; after: *M* = 26.05, *SEM* = 0.76; *F*_(1, 82)_ = 64.342, *p* < 0.001] and more negative [before: *M* = 11.45, *SEM* = 0.28; after *M* = 14.17, *SEM* = 0.54; *F*_(1, 82)_ = 26.205, *p* < 0.001]. There was no main effect for stimulation on positive [*F*_(3, 82)_ = 0.661, *p* = 0.579] or negative mood ratings [*F*_(3, 82)_ = 0.669, *p* = 0.573].

### Participants' ratings of laps picture stimuli

A significant effect was found for participants' valence ratings [*F*_(1, 81)_ = 705.030, *p* < 0.001]. Negative pictures [*M* = 2.14, *SD* = 0.89, rated on a 1–9 scale ranging from 1 “very negative” to 9 “very positive”] were rated more negative compared to neutral pictures (*M* = 5.64, *SD* = 73.). For arousal it was found that negative pictures (*M* = 6.97, *SD* = 1.45) were rated significantly more arousing than neutral (*M* = 4.19, *SD* = 1.29) pictures [*F*_(1, 80)_ = 268.681, *p* < 0.001].

### Side effects and blinding

Apart from a tingling sensation at the beginning of the session, no unpleasant side effects were reported. Blinding of stimulation condition was successful. Participants' conjecture of whether or not they receive tDCS during the task did not exceed chance level (see Table 2).

**Table 2:** **Perceived stimulation by participants in percent (%)**.

		**Actual stimulation**				
		**Total**	**Sham**	**0.5 mA**	**1 mA**	**1.5 mA**
	*N*	87	22	22	22	21
Perceived Stimulation	Sham	64.38	45.45	72.72	63.63	76.19
	Verum	35.62	54.55	27.27	36.36	23.81

### Behavioral data

In subjects that received sham tDCS no differences between negative and neutral distractor trials were found in respect to RT [*t*_(21)_ = 1.164, *p* = 0.258] and AC [*t*_(21)_ = −0.126, *p* = 0.901].

Analysis of the complete sample (sham, 0.5, 1.0, and 1.5 mA tDCS) revealed a main effect of valence on RT [*F*_(1, 83)_ = 3.943, *p* = 0.050]. Subjects responded slightly faster in trials with neutral pictures [*M* = 1151.04 ms, *SEM* = 20.254) than in trials with negative pictures (*M* = 1170.90 ms, *SEM* = 21.729, see Table 3). However, there was no significant effect of stimulation intensity [*F*_(3, 83)_ = 0.869, *p* = 0.461] and no interaction between picture valence and stimulation intensity [*F*_(3, 83)_ = 1.149, *p* = 0.334]. For AC, there was no main effect of valence [*F*_(1, 83)_ = 1.793, *p* = 0.184], no effect of stimulation intensity [*F*_(1, 83)_ = 1.05, *p* = 0.374] and no interaction between valence and stimulation intensity [*F*_(3, 83)_ = 0.137, *p* = 0.938].

**Table 3:** **Means of reaction time (RT) and accuracy (AC) in the DWM separately for stimulation intensity**.

	**Total**		**Sham**		**0.5 mA**		**1.0 mA**		**1.5 mA**	
***N***	**87**		**22**		**22**		**22**		**21**	
RT negative	1170.90	(21.73)	1135.21	(34.23)	1213.46	(42.21)	1164.88	(45.87)	1170.02	(51.81)
RT neutral	1151.04	(20.25)	1110.98	(34.62)	1206.59	(42.62)	1117.34	(38.04)	1170.09	(45.57)
AC negative	79.66	(0.76)	81.48	(1.49)	79.20	(1.61)	80.11	(1.78)	77.77	(1.02)
AC neutral	80.89	(0.81)	81.70	(1.74)	80.91	(1.66)	81.59	(1.43)	79.29	(1.72)

*RT measured in milliseconds, AC in percent (%). Standard error of the mean (SEM) is shown in brackets*.

### Electrophysiological data

**Figure 2:**
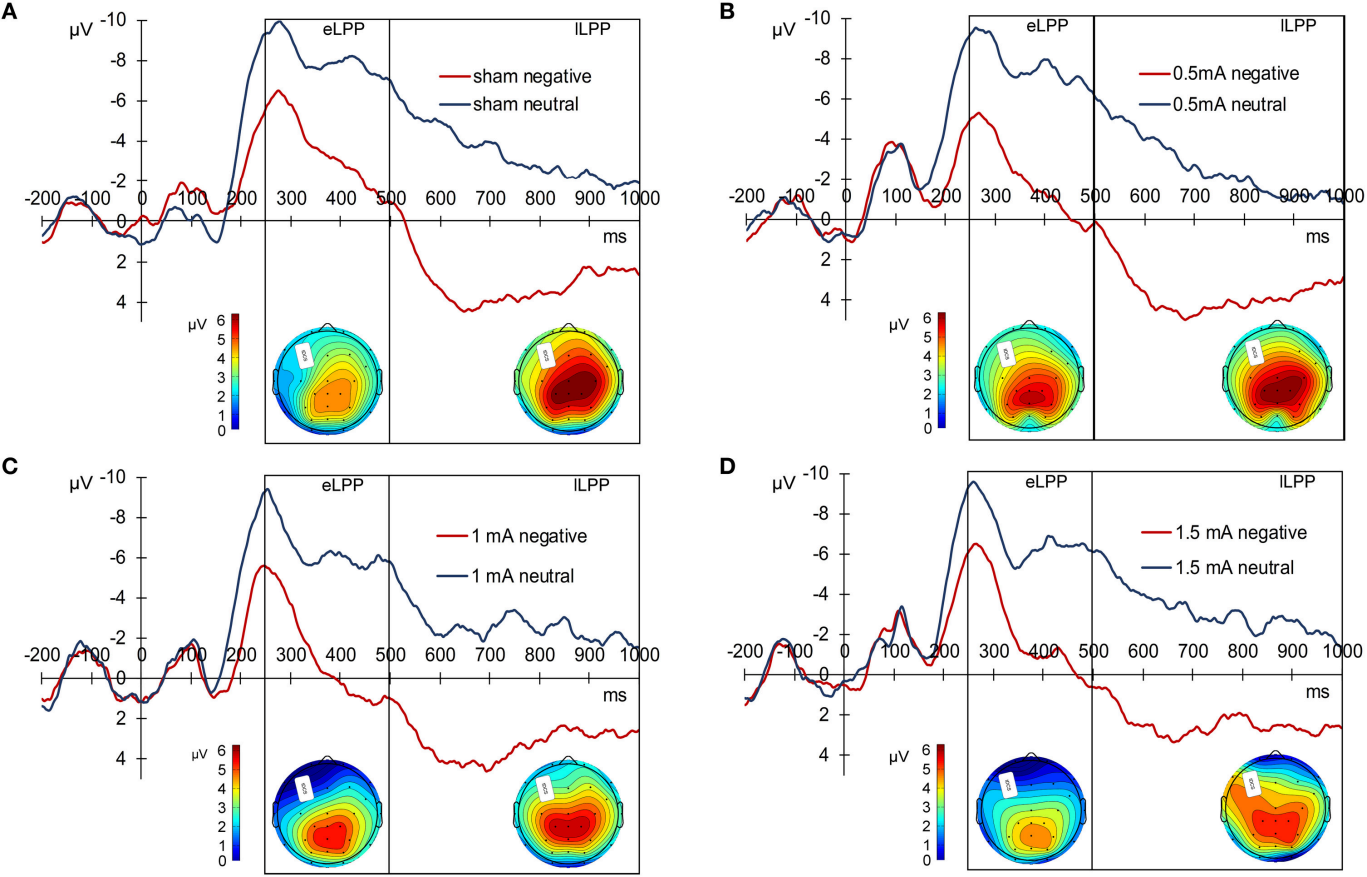
**Grand average LPP waves separately for the experimental conditions. (A)** Displays the sham, **(B)** the 0.5 mA, **(C)** the 1 mA, and **(D)** the 1.5 mA condition. Below each graph, there are two scalp maps displaying the mean voltage distribution for negative-neutral picture trials for the eLPP (250–500 ms) and lLPP (500–100 ms) time windows.

For statistical analysis, we divided the LPP in an early (250–500 ms, eLPP) and late (500–1000 ms, lLPP) window. There was a significant correlation between eLPP and lLPP for negative [*r*_(85)_ = 0.792, *p* < 0.001] as well as for neutral picture trials [*r*_(85)_ = 0.792, *p* < 0.001]. Figure 2 displays the grand average waveforms of the LPP for the sham (A), 0.5 mA (B), 1 mA (C), and 1.5 mA (D) experimental group and the mean voltage distribution across the scalp for negative-neutral picture trials for eLPP and lLPP.

### Early LPP (eLPP)

**Figure 3:**
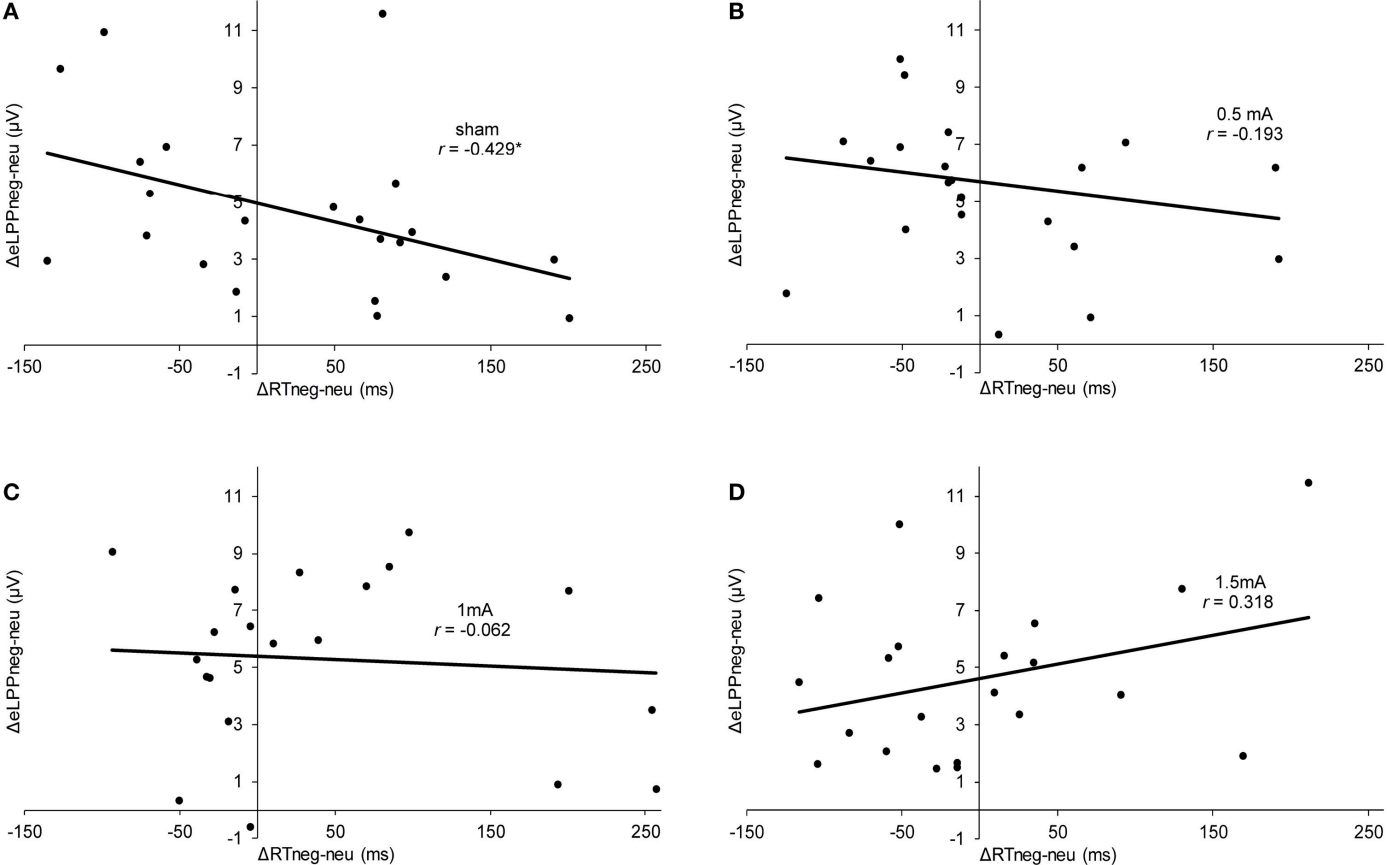
**Scatter plots illustrating the correlation between ΔRT_**neg-neu**_ and ΔeLPP_**neg-neu**_ in the sham (A), 0.5 mA (B), 1 mA (C), and 1.5 mA (D) experimental conditions**. In the sham group, the correlation was significant (^*^p = 0.046), while for the other experimental conditions no significant effect was found (0.5 mA:, *p* = 0.389;1 mA: *p* = 0.784; 1.5 mA: *p* = 0.160).

In the sham group, the difference between the eLLP amplitudes exerted by negative (*M* = −3.531, *SEM* = 1.192) and neutral pictures (*M* = −8.166, *SEM* = 1.002) was highly significant [*t*_(21)_ = 7.318, *p* < 0.001]. A negative correlation was found between RT and eLPP to negative [*r*_(20)_ = −0.512, *p* = 0.015] but not to neutral [*r*_(20)_ = −0.365, *p* = 0.148] stimuli. Consistently, a negative correlation [*r*_(20)_ = −0.429, *p* = 0.046, see Figure 3A] between valence specific brain activity (ΔeLPP_neg-neu_) and the distraction by negative stimuli (ΔRT_neg-neu_) indicated that the amount of additional eLPP activity elicited by negatively valenced pictures is linked with a less distractive or even beneficial influence of negative information on DWM performance.

Analysis of the whole sample again showed a higher eLPP amplitude associated with negative as compared to neutral pictures [*F*_(1, 83)_ = 230.141, *p* < 0.001, see Table 4]. However, no main effect of stimulation on the amplitude of eLPP [*F*_(3, 83)_ = 0.589, *p* = 0.624] was found and the valence by stimulation interaction was not significant [*F*_(3, 82)_ = 0.578, *p* = 0.631]. In the complete sample no correlation was found between RT and eLPP to negative [*r*_(85)_ = −0.184, *p* = 0.88] or neutral [*r*_(85)_ = −0.192, *p* = 0.75] stimuli. Correspondingly, no correlation between ΔeLPP_neg-neu_ and ΔRT_neg-neu_ was present neither in the whole sample [*r*_(85)_ = −0.94, *p* = 0.385] nor in the individual stimulation groups [0.5 mA: *r*_(20)_ = −0.193, *p* = 0.389; 1 mA: *r*_(20)_ = −0.062, *p* = 0.784; 1.5 mA: *r*_(19)_ = −0.318, *p* = 0.160; see Figures 3B–D]. But notably, adding stimulation to the task leads to a linear modulation of correlation coefficients with increasing intensities [*r*_(2)_ = 0.980, *p* = 0.020].

**Table 4:** **Mean amplitudes for eLPP and lLPP (in μV)**.

**Total**			**Sham**		**0.5 mA**		**1.0 mA**		**1.5 mA**	
***N***	**87**		**22**		**22**		**22**		**21**	
eLPP negative	−2.21	(0.64)	−3.53	(1.19)	−2.03	(1.34)	−1.07	(1.38)	−2.20	(1.24)
eLPP neutral	−7.26	(0.55)	−8.17	(1.002)	−7.68	(1.16)	−6.36	(1.06)	−6.83	(1.23)
lLPP negative	3.06	(0.55)	2.98	(0.98)	3.61	(1.26)	3.18	(1.15)	2.44	(1.09)
lLPP neutral	−2.97	(0.47)	−3.42	(0.89)	−2.63	(0.86)	−2.63	(0.92)	−3.21	(1.10)

*Standard error of the mean (SEM) is shown in brackets*.

### Late LPP (lLPP)

In the sham group, the lLPP amplitude was significantly higher (more positive) for negative (*M* = 2.979, *SEM* = 0.978) than for neutral (*M* = −3.423, *SEM* = 0.894) pictures [*t*_(21)_ = 9.305, *p* < 0.001]. The correlations between RT and lLPP to negative [*r*_(20)_ = −0.311, *p* = 0.159] and neutral [*r*_(20)_ = −0.365, *p* = 0.095] stimuli and between ΔlLPP_neg-neu_ and ΔRT_neg-neu_ [*r*_(20)_ = −0.274, *p* = 0.217] were not significant in the sham group.

For the whole sample, the amplitude of the lLPP following negative pictures was higher than for neutral picture trials [*F*_(1, 83)_ = 322.773, *p* < 0.001, see Table 4]. No significant effect of tDCS on the amplitude of lLPP was found [*F*_(3, 83)_ = 0.174, *p* = 0.914] and there was also no significant valence by stimulation interaction [*F*_(3, 83)_ = 0.272, *p* = 0.845]. However, across all participants, RT and lLPP were significantly correlated negatively both for negative [*r*_(20)_ = −0.259, *p* = 0.015] and neutral picture trials [*r*_(20)_ = −0.254, *p* = 0.018]. Correspondingly, for ΔRT_neg-neu_ and ΔlLPP_neg-neu_, no relationship was found in the complete sample [*r*_(85)_ = −0.077, *p* = 0.479] and any of the stimulation conditions [0.5 mA: *r*_(20)_ = −0.286, *p* = 0.197; 1 mA: *r*_(20)_ = −0.061, *p* = 0.789; 1.5 mA: *r*_(19)_ = 0.425, *p* = 0.285].

## Discussion

In this study, we explored the neurophysiological signatures of cognitive control on emotional distraction and the intensity-dependent influence of tDCS by parallel ERP/tDCS recordings in a delayed working memory task (DWM) with negative and neutral distractors. Key findings are that (i) without tDCS, higher valence-specific neuronal activation as indicated by the early phase of the LPP (ΔeLPP_neg-neu_) is associated with less distraction by negative pictures (ΔRT_neg-neu_), (ii) tDCS exerts an intensity-dependent influence on this correlation, and (iii) a stimulation and valence-independent correlation is present between the later phase of the LPP (lLPP) and RT performance. Further effects of tDCS on WM performance, distraction by emotional stimuli or LPP amplitudes could not be identified.

It has already been demonstrated that the LPP can reflect both automatic responses to salient stimuli as well as higher cognitive control mechanisms (Olofsson et al., 2008; Bamford et al., 2015). Therefore, it has been proposed to examine the LPP in multiple time windows (Foti et al., 2009; Hajcak et al., 2010b). In our study, we selected two time windows of the LPP which we hypothesized to have differential relations to RT performance under emotional distraction. For the early LPP (eLPP, 250–500 ms) elicited by negative pictures and correspondingly for valence specific ΔeLPP_neg-neu_, we found a correlation with performance (RT and ΔRT_neg-neu_) in the group not subjected to tDCS. For the late LPP (lLPP, 500–1000 ms) we demonstrated a significant correlation for both neutral and negative trials in the complete sample.

In a nutshell, our findings in the sham stimulation group suggest the recruitment of additional neuronal activation as signified by a higher eLPP amplitude to compensate the influence of distracting negative information allowing for goal-directed performance. The corresponding correlation between ΔRT_neg-neu_ and ΔeLPP_neg-neu_ demonstrates that the presentation of negative pictures during the maintenance phase of the WM tasks can, in different subjects, both interfere and enhance performance. According to Figure 3A, subjects with a mean negativity-related eLPP increase of less than 5 μV (as indicated by the y-intercept) were distracted by negative pictures but those with a larger valence-specific eLPP increase reacted faster in trials with negative pictures. These findings are in agreement with the existing literature on emotion-cognition interaction. It is well known that emotion can facilitate ongoing task performance by recruiting common resources, which is especially true for low-threat stimuli (Pessoa, 2009) and that emotion may facilitate task performance (for a review see Okon-Singer et al., 2015). For instance, Wessa et al. (2013) found augmented task related activation in the presence of distracting emotional stimuli in cerebral regions that were identified to be task relevant. Using the LPP as a neuronal marker, Bamford et al. (2015) studied the influence of emotional stimuli in an approach avoidance paradigm. Pictures of negative or neutral content were presented and participants had to either “approach” or “avoid” these by pressing buttons. Here, larger LPP amplitudes were associated with faster RTs, regardless of the condition of the task. Moser et al. (2010) found that the instruction to increase the emotional response to a negative picture was associated with a higher LPP amplitude and a significant improvement in a subsequent cognitive control (“Stroop”) task. Together with these findings, our results add evidence that the LPP also indexes processes which are necessary for task performance in behavioral tasks involving emotional information. *Prima facie*, this contrasts with previous findings suggesting that high LPP amplitudes indicate high attentional involvement with a distractive stimulus resulting in poorer task performance (Weinberg and Hajcak, 2011). However, it has to be considered that in our study the distractor was presented not before but during memory maintenance. Therefore, the beneficial effect of enhanced activation as reflected by a larger eLPP shown in our study is actually consistent with the concept of an additional recruitment of executive processes by emotional content (González-Garrido et al., 2015). Remarkably, this process can, at least in some subjects, lead to an enhanced RT performance by distractive negative stimuli.

Regarding the lLPP we found a comparable correlation between amplitude and RT performance but in this case unaffected by the emotional content (i.e., lLPPs elicited by negative and neutral pictures were both positively correlated with RT-performance and emotion-specific activity (ΔlLPP) and RT (ΔRT) were not correlated). This finding further exemplifies that the amount of brain activation associated with distractive pictures during the maintenance phase of a WM task is critical for performance. However, these data are also consistent with evidence that brain activity reflected by the lLPP is affected by cognitive demand, for instance memory load (MacNamara et al., 2011; Van Dillen and Derks, 2012; Schönfelder et al., 2014), with higher load being associated with lower LPP amplitudes, both for negative and neutral distractive pictures. As we used an inter-subject design, our findings may be attributed to the inter-individual variability in WM capacities. Relatively low individual WM load might be reflected by shorter RT and associated with larger lLPPs to distractive pictures. In turn, relatively high individual WM load presenting with longer RTs are linked with lower lLPPs. Since the attenuating effect of WM load has been demonstrated with positive as well as negative distractors (MacNamara et al., 2011), it is consistent that in our data emotional valence has no differential influence.

In respect to the effect of tDCS, we expected a modulation of LPP amplitudes and an influence on the negativity bias by stimulation. However, this hypothesis was not supported by our data. Stimulation with different intensities of anodal tDCS did not yield a systematic effect on behavior or LPPs. Especially regarding the LPPs, the generally high inter-individual variability may have precluded the detection of systematic effects of stimulation particularly by means of the between-subject design applied in this study. This is important to note since previous studies showing effects of tDCS on the negativity bias applied a within-subject design that reduces the influence of inter-individual variability (Vanderhasselt et al., 2013; Wolkenstein and Plewnia, 2013; Wolkenstein et al., 2014). Next, a plausible explanation for the lack of significant effects is the rather weak negativity bias that was detected in the complete sample but not in the sham group alone. Naturally, an absent bias cannot be ameliorated. As this study was conducted with healthy participants, we tried to use highly negative pictures to induce a depression-like negativity bias (Wolkenstein and Plewnia, 2013). However, the only minimal distraction by negative pictures suggests that healthy subjects are mostly able to compensate for the influence of these stimuli. Accordingly, adding anodal tDCS to basically intact cognitive control functioning seems to rather add noise to a well-balanced system without inducing meaningful effects. Moreover, in dependence of the individual conditions, anodal tDCS might have even preferentially enhanced performance under emotionally neutral distraction and thus induced a better performance in neutral as compared to negative conditions (Wolkenstein and Plewnia, 2013). Actually, the significant parametric change of the correlation between ΔRT_neg-neu_ and ΔeLPP_neg-neu_ by increasing stimulation intensity suggests that anodal tDCS might modulate or even reverse the association between negativity bias and valence specific brain activity in healthy subjects. Of note, being independent from emotional content, the association between lLPP and RT has not been influenced by tDCS, pointing toward a preferential modulation of emotion-related cognitive control processes by prefrontal tDCS.

With the parallel tDCS/ERP recording, our study provides important insights in the feasibility of this rather novel approach (Cunillera et al., 2015). The EEG acquisition parallel to tDCS was unproblematic and data quality was not impaired. However, due to artifacts significantly more ERP trials had to be rejected in the 1.5 mA group as compared to sham stimulation. Still, this is not likely to affect the results presented since the LPP has been shown to be very robust to loss of trials (Moran et al., 2013) and loss was still minor. Remarkably, stimulation with a maximum 1.5 mA was possible with successful blinding of participants. To achieve good recording quality, we had to remove electrodes surrounding the tDCS electrode. Moreover, using conductive paste instead of sponge electrodes seemed to help to minimize interactions between tDCS and EEG.

Limitations of the study are first that, to warrant practicability, only highly arousing negative stimuli were used. This is in general agreement with the notion that the LPP is modulated particularly by arousing stimuli (Codispoti et al., 2006; Hajcak et al., 2013). It is actually possible that highly arousing positive pictures will yield comparable results. Second, to increase the homogeneity of our sample we only included female participants. Nevertheless, it would be interesting to assess the influence of gender, as it has previously been shown that attention allocation toward emotional stimuli as mirrored by the LPP differs with gender (Syrjänen and Wiens, 2013). Third, concerning the time windows of the eLPP and lLPP, the available literature provides variable definitions (Hajcak et al., 2010b; Wessing et al., 2015). We decided to examine one early and one late window due to evidence that modulation by valence was predominantly found in the earlier phase (Codispoti et al., 2007) and refrained from analysis beyond the 1000 ms range because previous research indicated attention modulation to be predominantly important in the first second (Moser et al., 2014).

In sum, with this study we demonstrated that tDCS/ERP recording is a feasible method to track online effects of stimulation. The findings support the notion of the LPP as a neuronal marker for cognitive control, measured by RT performance in a WM task with emotional distractors. Furthermore, they provide evidence that the LPP amplitude induced by a distractive stimulus mirrors allocation of neuronal resources that support task performance. Particularly, the emotion specific increment of its early portion (eLPP), signals effective compensation for behavioral distraction by negative stimuli and thus points toward a neuronal mechanism for effective control of the emotional bias. In contrast, the association of the later phase (lLPP) with RT is not emotion specific. Finally, no systematic stimulation effects on LPPs and performance were found. Considering the inter-individual differences in magnitude and direction of the emotional bias, indicating highly variable activation of cognitive control networks, these results underline the critical interaction between brain activity and tDCS effects.

## Author contributions

CP and FF designed study, FF collected data, FF and CP analyzed data, FF and CP wrote to the manuscript.

### Conflict of interest statement

The authors declare that the research was conducted in the absence of any commercial or financial relationships that could be construed as a potential conflict of interest.

## Appendix

(A) Text of the instructions preceding the DWM task:

Next, you will see a cross in the middle of the screen. Please look at it. Then eight letters will appear. Please memorize these letters. Then a picture will appear and afterwards one single letter. If you believe that the single letter already appeared in the eight previous letters, please press the J key. If you believe that it did not appear, press the F key. Hold your index fingers on the keys for the whole time of the experiment. Please answer as quickly as possible. In between, there will be breaks of 15 s. Among the pictures, there will be very unpleasant ones, including dead human bodies. Please do not close your eyes. If you want to end the experiment at any time, just tell the supervisor. Try to relax your face and sit relatively still. Do you have any questions?

(B) Index numbers of pictures taken for the DWM from the IAPS database:

Negative pictures: 2703,2800,3001,3010,3015,3016,3030,3051, 3053,3059,3060,3064,3071,3100,3102,3110,3120,3130,3131,3140, 3150,3230,3261,3350,3400,3530,3550,6313,6315,6350,6510,9040, 9253,9250,9253,9265,9405,9410,9433,9412.

Neutral pictures: 2025,2038,2039,2102,2190,2191,2200,2210, 2214,2215,2221,2235,2270,2272,2305,2372,2374,2383,2400,2411, 2487,2490,2493,2500,2512,2514,2575,2579,2590,2595,2749,2745.1,2840,2850,2870, 2890,7493,7503,7505,9210.

Training pictures: 5455,7130,7140,7180,7234,7490,7491,7496,7700.
